# Topical Gene Electrotransfer to the Epidermis of Hairless Guinea Pig by Non-Invasive Multielectrode Array

**DOI:** 10.1371/journal.pone.0073423

**Published:** 2013-08-28

**Authors:** Siqi Guo, Annelise L. Israel, Gaurav Basu, Amy Donate, Richard Heller

**Affiliations:** Frank Reidy Research Center for Bioelectrics, Old Dominion University, Norfolk, Virginia, United States of America; University of Nebraska Medical Center, United States of America

## Abstract

Topical gene delivery to the epidermis has the potential to be an effective therapy for skin disorders, cutaneous cancers, vaccinations and systemic metabolic diseases. Previously, we reported on a non-invasive multielectrode array (MEA) that efficiently delivered plasmid DNA and enhanced expression to the skin of several animal models by *in vivo* gene electrotransfer. Here, we characterized plasmid DNA delivery with the MEA in a hairless guinea pig model, which has a similar histology and structure to human skin. Significant elevation of gene expression up to 4 logs was achieved with intradermal DNA administration followed by topical non-invasive skin gene electrotransfer. This delivery produced gene expression in the skin of hairless guinea pig up to 12 to 15 days. Gene expression was observed exclusively in the epidermis. Skin gene electrotransfer with the MEA resulted in only minimal and mild skin changes. A low level of human Factor IX was detected in the plasma of hairless guinea pig after gene electrotransfer with the MEA, although a significant increase of Factor IX was obtained in the skin of animals. These results suggest gene electrotransfer with the MEA can be a safe, efficient, non-invasive skin delivery method for skin disorders, vaccinations and potential systemic diseases where low levels of gene products are sufficient.

## Introduction

Epidermal gene transfer is suggested as a new therapeutic strategy for a variety of skin diseases, vaccination and systemic disorders [1]–[5]. Long-term or persistent gene delivery to the epidermis has promise for inherited skin diseases and potentially systemic disorders [3], [5]. On the other hand, short-term epidermal gene delivery is suitable for vaccination, skin wound or ulcer therapies and skin malignancy [2]–[6]. Topical application of plasmid DNA results in low levels and short duration of gene expression in epidermal skin [7]. Cutaneous gene electrotransfer (GET) following intradermal DNA injection has been widely studied [8]. The level and duration of gene expression is quite different, depending on which type of electrodes and which species of animals was utilized [8]–[14]. However, definitive epidermal expression by GET in guinea pig or human skin xenograft mouse model has been demonstrated by only a few groups of researchers [9], [12], [15], [16].

The human skin xenograft mouse model may be the best choice for preclinical skin research [17]–[19]. However, sources of human skin are very limited. A second option is hairless guinea pig (HLGP) skin, whose skin is very similar to human skin anatomically and histologically as opposed to the skin of normal rodents [20]. Previously, we [12], [16] demonstrated that GET to the skin resulted in efficient expression in rat and the Hartley guinea pig (haired). In this study, we further characterize several important aspects associated with potential clinical applications, such as the level and duration of gene expression, distribution of gene expression within the tissue and side effects in this model. We also investigated if a therapeutic protein, human factor IX, could be expressed in the skin following delivery using the non-invasive MEA and whether it could reach the blood circulation.

## Materials and Methods

### Animals

Female IAF hairless guinea pigs (Charles River, Wilmington, MA, USA) used in this study were 160 g to 200 g in weight. All experimental procedures were approved by the Institutional Animal Care and Use Committee of Old Dominion University. All treatment was performed under isoflurane anesthesia, and all efforts were made to minimize suffering.

### Plasmids

The reporter plasmids encoded luciferase (gWiz-Luc) and green fluorescent protein (gWiz-GFP), were acquired from Aldevron (Fargo, ND, USA). Human Factor IX expression vector pNGVL3/CMVi/hFIXm1given by Dr. Kurachi was commercially prepared by Aldevron. All three plasmids are with a CMV promoter.

### DNA Injection and *in vivo* Gene Electrotransfer

Prior to delivery, animals were anesthetized in an induction chamber charged with 3% isoflurane in O_2_ then fitted with a standard rodent mask and kept under general anesthesia during the procedure. Guinea pigs received intradermal injections of 50 µl or 200 µl plasmid DNA (2 µg/ µL dissolved in saline) on the left and/or right flank. Immediately after DNA administration, a MEA electrode with 4×4 2-mm-apart pins was placed over the injection site(s). Each pair of electrodes was programmed to administer four pulses with total 72 pulses [16]. The applied voltage varied between 30–70 V between the two pins of the MEA which was set at 2 mm to achieve nominal field strengths between 150 – 350 V/cm, each pulse duration was 150 ms with a 150 ms delay. Electrotransfer was performed using the UltraVolt Model: Rack-2-500-00230 (Ultravolt, Inc. Ronkonkoma, NY, USA). For four 50 µl adjacent injections, four individual pulse applications were applied without a change of pulse parameters. No conductive gel was used. A flexible spring is placed in the substrate of each pin of MEA to assure a full contact between the uneven skin surface and all of the electrodes. The delivery parameters such as pulse numbers, electric field and current were monitored.

### Living Imaging of Luciferase Expression

At different selected time points after delivery, animals were anesthetized then administrated intradermally with 50 µL or 200 µLof D-luciferin with 7.5 mg/mL in PBS buffer (Goldbio, St. Louis, MO, USA). Assessment of photonic emissions using the IVIS Spectrum system (Caliper Life Sciences, Hopkinton, MA, USA)) was performed 1.5 minutes after injection of D-luciferin. Background luminescence was determined by measuring luminescence from area without DNA injection and electric field.

### GFP Expression

Each excised sample was immediately frozen on dry ice. After visualization of GFP expression was observed and obtained by fluorescent stereoscope (Leica Model MZFL III, Leica, Heerbrugg, Switzerland), the specimens were embedded in tissue freeze media OCT compound (Electron Microscopy Sciences, Hatfield, PA) and frozen at −80°C. Several frozen sections (8 µm thickness) were cut from each sample. Each section was fixed in 25% acetone in ethanol for 20 minutes and then washed twice in PBS. Sections were dried in the dark and the coverslip mounted with VECTASHIELD® mounting medium with DAPI (Vector Laboratories, Burlingame, CA). Sections were examined by Olympus BX51 fluorescent microscopy (Olympus, Tokyo, Japan) for the presence of GFP.

### Histological Analysis

Each specimen was embedded, sectioned and fixed as mentioned above. Sections were dehydrated in 95% ethanol 30 seconds, stained in hematoxylin solution 5 minutes, rinsed with tap water 3 minutes, classified in 1% acid alcohol for 10 seconds, washed with running tap water for 1 minute, blued in 0.2% ammonia solution for 30 seconds, washed in running tap water for 3 minutes, rinsed in 95% alcohol, 10 dips, counterstained in eosin Y solution for 45 seconds, dehydrated through 95% alcohol, 2 changes of absolute alcohol, 10 dips each, cleared in 2 changes of xylene, 10 dips each, mounted with xylene based mounting medium. Sections were examined by Olympus BX51 microscopy.

### ELISA assay for human factor IX

Skin samples were harvested and immediately frozen on dry ice at different time points after EP delivery. The supernatants were collected after skin homogenization in PBS with protein inhibitor (Cat. 04693132001, Roche Applied Science, Mannheim, Germany). At different time points, 400 µL blood were collected in serum collecting tubes with 50 µL 0.5 M EDTA and plasma collected after centrifugation. To perform an ELISA assay, a Nunc 96-well ELISA plate was coated with mouse anti-hFactor IX antibody (Sigma Aldrich, St. Louis, MO, USA) at 200 ng/well in PBS and incubated at 4°C overnight. Human factor IX (Promega, Fitchburg, WI, USA) was serially diluted as a standard. HRP-goat anti-hFactor IX (1∶2000, Enzyme Research Labs, South Bend, IN, USA) was the detection antibody. Substrate reagent (R&D System, Minneapolis, MN, USA) was added and finally 1N sulfuric acid stop solution was added. The plate was read by Multiskan MCC Microplate Reader (Fisher Scientific) and the concentration of factor IX was determined using a standard curve.

### Statistical Analysis

All values are reported as the mean ± SD. Area under curves (AUCs) for luciferase or human factor IX expression are calculated for analysis of gene expression over the time among different delivery groups. The linear trapezoidal method is used for the rising phase of gene expression while logarithmic trapezoidal method is used for the decreasing phase of expression. Analysis was completed by One Way ANOVA for many groups or 2-tailed Student's t-test for 2 groups. Repeated Measures ANOVA was used to evaluate the differences of animal weight increase after treatment. Statistical significance was assumed at p<0.05. All statistical analysis was completed using the SigmaPlot 11.0 (Aspire Software International, Ashburn, VA).

## Results

### Significant elevation of gene expression and prolongation of expression after intradermal DNA administration followed by topical non-invasive skin GET

**Figure 1:**
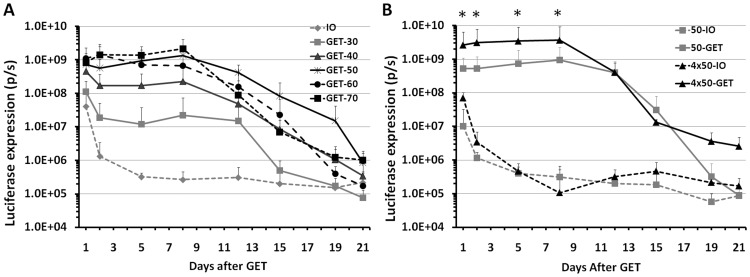
Kinetic of gene expression in HLGP skin after intradermal DNA injection and non-invasive GET. **A**, Time course of luciferase expression in HLGP skin after delivery by different electric fields at day 0. 50 µL DNA and 1 pulse delivery for all delivery groups, IO: no pulse delivery; GET: applied voltage of 30, 40, 50, 60 or 70. *: p<0.05 for GET-50, GET-60 or GET-70 vs IO. **B**, Time course of luciferase expression in HLGP skin after delivery to different sizes of skin. Delivery groups, 50-IO: 50 µL DNA without pulse delivery; 50-GET: 50 µL DNA with 1 pulse delivery on the injection site; 50 µLx4-IO: 4 injections with 50 µL DNA without pulse delivery; 50 µLx4-GET: 4 injections with 50 µL DNA and each pulse delivery on the injection site. Bars represent mean ± SD. 4–6 sites were analyzed for each delivery parameter, p/s  =  photons/second. * p<0.05 for 4×50-GET vs 50-GET and 4×50-GET vs 4×50-IO.

Because electrogene delivery had not been previously performed in hairless guinea pig skin, we first optimized the delivery condition by adopting the parameters from our previous work in Hartley guinea pigs [12], while testing a wider range of electric fields. As shown in Fig 1A, the expression level of luciferase positively correlated to the applied voltage up to 50 V. Although all delivery groups with above 50 V showed significantly increased gene expression compared to DNA injection only (p<0.05 for all 3 groups vs DNA injection only), the level of gene expression was no longer enhanced when the applied voltage was further increased up to 70 V. In contrast to intradermal DNA injection only, GET remarkably enhanced gene expression 2 to 4 logs and prolonged gene expression up to 12 to 15 days. While the expression level of non-GET-treated groups was relatively low at day one and rapidly dropped at day two, the gene expression of GET-treated groups maintained high levels until peak expression was reached at day eight then slowly decreased to background level after day 19 or later.

We next addressed the feasibility of increasing the gene expression by the extension of the treated areas. We compared a 50 µL DNA injection with a single GET application to a four-fold increase in plasmid and delivery area, This resulted in a 3.94 to 6.02 fold increase in luciferase from days one to eight after delivery (Fig 1B, p<0.05 for 4 GET vs 1 GET). Eight days after electrogene delivery, gene expression in both groups gradually decreased. However, at day 21 post-delivery, the gene expression in the group treated in the larger area maintained 1 log higher than the control group without GET. For the group receiving the single GET application, gene expression was similar to the respective control group.

### Exclusively epidermal gene expression after topical DNA delivery with a noninvasive MEA

**Figure 2:**
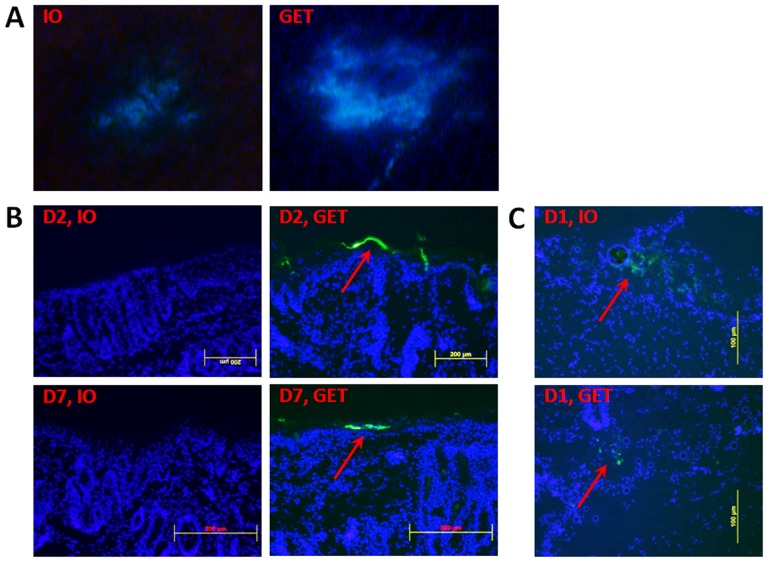
Distribution of GFP-expressing cells in HLGP skin after i.d. DNA injection and non-invasive GET. Skin samples were collected post-delivery day 1, day 2 or day 7. Samples were analyzed by fluorescent stereoscopy or microscopy. IO: DNA injection only without pulse delivery; GET: DNA injection with pulse delivery. **A**, One representative picture of 3 treated sites. (B, C) Total 6 cryosections (2 sections per sample) of each delivery were analyzed. Cell nuclei were blue-stained by DAPI. GFP-expressing cells were shown green and indicated by arrow. **B**, One representative section of each delivery was presented for post-delivery day 2 or day 7(magnification  = 100, scale bar = 200 µm). **C**, One representative section from post-delivery day 1 (magnification  = 200, scale bar = 100 µm).

Using fluorescence stereoscopy, a weak and small area of green florescence protein (GFP) expression was present at the center of injection site in non-electrotransferred skin one day after intradermal DNA injection alone. In contrast, GET enhanced the gene expression with increase of not only the intensity of gene expression but also expression area (Fig 2A), which was slightly larger than the surface covered by MEA. To assess the distribution of transgene expressing cells after non-invasive surface GET, cross-sections of the skin were labeled with DAPI and observed by fluorescent microscopy. All GFP expressing cells from GET-treated skin were located in the epidermis at post-GET delivery day 2 or day 7 (Fig. 2B). For skin receiving DNA injection alone, no expression was observed in the epidermis at either day 2 or day 7 (Fig. 2B). The distribution pattern of transgene expressing cells is the same as we observed in Hartley guinea pigs [12] although their skin histologies are not identical. As previously mentioned, skin receiving DNA injection only expressed a low level of luciferase and GFP one day after gene delivery (Fig. 1 and Fig. 2A). In fact, a few GFP expressing cells in skin without pulse delivery were observed in the dermis surrounding the DNA injection site but not in the epidermis (Fig. 2C). It appears that epidermal gene expression only resulted from GET.

### Minimal and mild skin changes after GET with a noninvasive MEA

**Figure 3:**
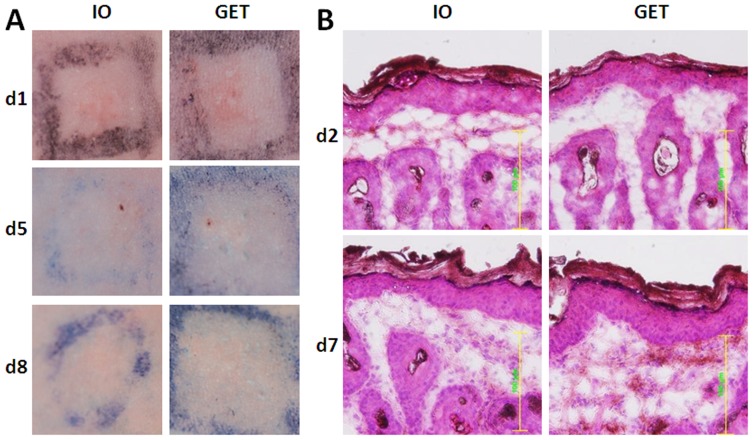
Gross observation and histology of HLGP skin after i.d. DNA injection and non-invasive GET. **A**, Skin observation after delivery. Pictures were taken at post-delivery day 1, day 5 and day 8. One representative picture of 4 to 6 sites was shown here. Delivery group, IO: 50 µL DNA without pulse delivery; GET: 50 µL DNA with pulse delivery. **B**, H&E stained skin samples. One representative of 3 treated sites was presented here for post-delivery day 2 or day 7 (magnification  = 200, scale bar = 100 µm).

Another important issue is the adverse effect of GET with the non-invasive MEA. Based on gross observation, GET with parameters selected in this study did not cause any severe tissue damage such as skin burning, necrosis or scar formation (Fig. 3A). Muscle twitch also was greatly reduced with this non-invasive MEA compared to plate electrode or needle array. Skin redness remained one day after GET delivery, but faded by day 2. Only the needle track at the injection site was seen at post-delivery day 5 (Fig. 3A). Tissue damage was also evaluated by hematoxylin and eosin staining. Microscopically, no significant morphological changes at day 2 or day 7 were observed in skin with GET treatment (Fig 3B). The focal cell vacuolization or degeneration of the skin, which was seen in the epidermal layer in our previous study with Hartley guinea pigs (Heller *et al.* 2007), was not observed in this study. Overall, these skin changes with gross observation and histology were milder than Hartley guinea pig skin treated with same GET parameters.

**Figure 4:**
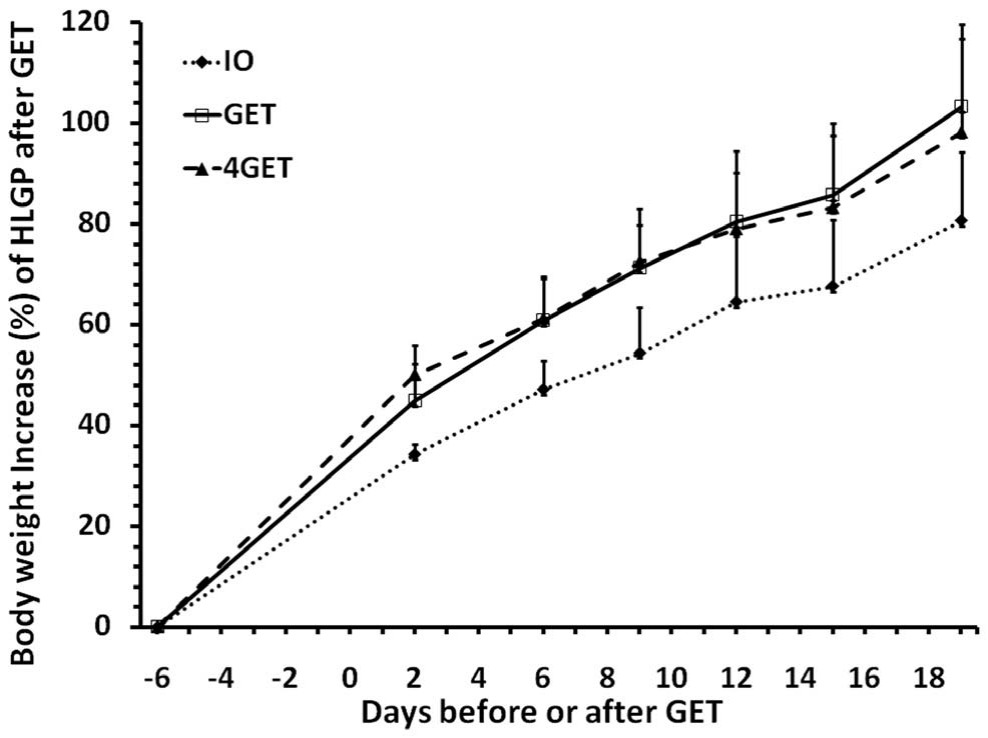
Body weight increase of HLGP after i.d. DNA injection and non-invasive GET. Delivery group, IO: 50 µL DNA without pulse delivery; GET: 50 µL DNA with 1 pulse delivery on the injection site; 4GET: 4 injections with 50 µL DNA and each pulse delivery on the injection site. Bars represent mean ± SD. 4 animals were analyzed for each delivery group. * p<0.05 for 4GET or GET vs IO by One Way RM-ANOVA.

The average body weight increase of plasmid and GET treated HLGPs was 10.7% to 22.7% greater than those without GET treatment in our observational period from day 2 to day 19 (Fig 4, p<0.001 for GET or 4 GET vs DNA alone control). The change of body weight appears related to GET only but not the size of GET treatment. Increasing delivery to four areas did not further enhance the level of body weight increase (Fig. 4), although it was observed to further significantly increase transgene expression (Fig. 1a).

### Human factor IX production in the HLGP skin and secretion into systemic circulation after GET to the skin with the non-invasive MEA

**Figure 5:**
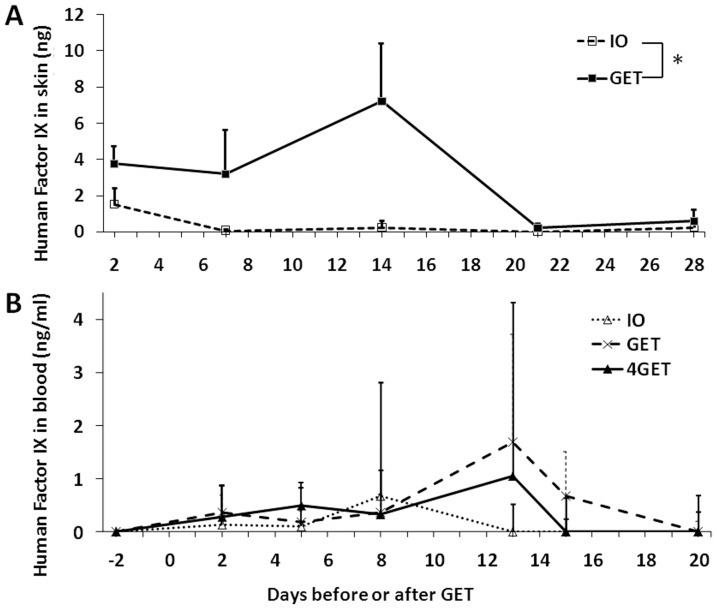
Human factor IX protein in HLGP after i.d. DNA (pNGVL3/CMVi/hFIXm1) injection and non-invasive GET. **A**, Human factor IX in HLGP skin. Delivery groups, IO: 50 µL DNA without pulse delivery; GET: 50 µL DNA with 1 pulse delivery on the injection site. Bars represent mean ± SD. 3 sites were analyzed for each delivery. * p<0.05 by Student's t-test. **B**, Human factor IX in HLGP plasma. Delivery groups, IO: 50 µL DNA without pulse delivery; GET: 50 µL DNA with 1 pulse delivery on the injection site. 4GET: 4×50 µL DNA with 4 pulse delivery on the injection site. Bars represent mean ± SD. 4 HLGPs were analyzed for each delivery group.

Topical expression of a therapeutic protein, human factor IX, was next assessed by our established approach. A significant level of human Factor IX protein in HLGP skin was achieved with electrotransfer (Fig 5A, p = 0.036). Three weeks after delivery, no significant factor IX product was detected. In contrast, plasmid DNA injection alone showed expression of factor IX only at day 2 of 1.5 ng/delivery area. Several groups have demonstrated the expression of human factor IX or growth hormone in mouse epidermis could reach the systemic circulation [21]–[23]. Here we observed if our direct topical electrotransfer approach could do the same. Plasma was collected at different time points before and after topical gene delivery and analyzed by ELISA. No increase of factor IX protein in HLGP plasma was observed after DNA injection only. Although a low level increase of factor IX protein, 0.2 ng/ml to 1.7 ng/ml, was observed from days 2 to 15 after electrotransfer (Fig 5B), there is no statistically significant difference among control and delivery groups.

## Discussion

The xenograft human skin-SCID chimera has been reported as a valuable model for pathological research of several skin disorders including psoriasis, cutaneous lupus, pemphigus, vitiligo and androgenetic alopecia [24]–[26]. However, the immune deficiency of the xenograft model limits its application for immunomodulation, including vaccination and immune therapy for skin cancer. A few skin electrotransfer studies have been performed in rabbit or pig skin, while most studies of skin GET have been performed in mouse or rat skin [8], which is significantly different from human skin. The structure of HLGP skin, including thickness, histology and number of microvessels, is very similar to human skin [20]. In addition, these animals are euthymic and immunocompetent, which makes HLGP skin a good alternative for human skin research. A major disadvantage of the HLGP model is the unavailability of antibodies for identification of specific cell types.

Our contact electrode (MEA) is one of the most suitable systems for delivery to the skin epidermis. Skin GET can result in transfected cells in the epidermis, dermis, hypodermis, and even the muscle layer, dependent on the electrodes, injection techniques, animal species and plasmid designs used, for example whether tissue specific promoters are involved. GET with plate electrodes transfects cells to the mouse dermis [27], the epidermis of xenograft human skin [9], or both epidermis and dermis of rat [13]. GET with needle electrodes or needle arrays can reach the deep layers of the skin and result in transgenic cells in the dermis, epidermis, hypodermis and subcutaneous muscle layer of the mouse or rat [14], [28] or dermis of the pig [29]. Interestingly, both epidermis and muscle layer were transfected by pulse delivery with plate-and-fork electrode as was shown with a variation of the time-course of expression [11]. Moreover, transfection in the lower dermis of rabbit was achieved by GET with tweezer electrodes [30]. In this study and in our previous work [12], we demonstrated that targeted transgenic expression to the epidermis can be obtained in Hartley guinea pig or HLGP skin after GET with the MEA. A similar result was observed in another study performed in Hartley guinea pig skin using a similar, but more invasive electrode [15]. The computer simulation of the skin model for delivery with the meander electrode showed that majority (>90%) of electric field acted in the epidermal layer of skin within a depth of 125 µm [31]. It is understandable that GET with surface contact electrodes like the MEA results in exclusively transgene expression in the epidermis.

Several studies have reported the expression level and duration of gene delivery with skin GET [8]–[14], it is hard to make an accurate comparison among them because different animal species were used and delivery of various transgenes was performed using a variety of pulse parameters and electrodes. Obviously, gene transfection into different cell types can lead to various levels and kinetics of transgenic expression because the cell half-lives are varied. In addition, the variation in half-lives of gene products can contribute to the duration of expression. It is well known that transfection of stable cells such as muscle fibers can result in relatively high and long-term gene expression, while transfection of fast growing cells such as cancer cells produces a very short duration of gene expression. Skin GET with a plate electrode either in mice or rats can produce transgene expression with duration of two weeks [10], [13], which is similar in time course to our studies with guinea pig or HLGP skin with MEA GET. However, the patterns of transgene expression are different between GET with a plate electrode and GET with the MEA. The former results in a spike of expression at days 1 to 2 in the rat or day 9 in the mouse while the latter maintains a high level expression for 8 to 12 days. The difference of expression patterns can be explained by the distribution of the transfected cells. GET with plate electrode will transfect both epidermal and dermal cells, while GET with the MEA will only transfect into epidermal cells. If the total amount of expression or the area under curve is determined, delivery with the MEA achieves a higher total expression.

Compared to other skin GET, such as plate or needle electrodes, the adverse effects caused by MEA were largely reduced. Muscle contraction caused by GET was decreased, and no serious skin damage was observed grossly and histologically. We observed that the side effects were similar or milder in HLGP than Hartley guinea pigs or rat treated by MEA with same parameters[12], [16]. Noticeably, significant weight gain was related to skin GET with MEA for HLGP. We haven't observed this phenomenon on Hartley guinea pigs or rat under same condition. Because of small sample size, only 4 animals per group, we don't know if it's real. To confirm this result, we will recommend it's necessary to repeat the same experiment with large group of animals.

Previously, we demonstrated that gene expression after GET with the MEA was significantly higher than GET with a plate electrode in the Hartley guinea pig model [16]. Using the same electrical parameters, the expression levels achieved by GET with the MEA were different depending upon the animal species. Among rat, Hartley guinea pig and HLGP electrotransfer with the MEA would achieve the highest gene expression in HLGP skin, then in the Hartley guinea pig skin [12], the relative lowest gene expression in rat [16]. The thicker epidermis of HLGP skin is most likely related to its higher expression level. Because of the similarity of histology and thickness between HLGP skin and human skin we would predict that human skin with the MEA can achieve the same level of gene expression under the similar electrical parameters.

Another important question is whether the protein produced in the epidermis following GET can reach the blood circulation of the animals and achieve a therapeutic level. In this study we demonstrated a low level of human Factor IX (0.2 ng/ml to 1.7 ng/ml) could be detected in the HLGP circulation if a large area of skin is GET-treated. Human factor IX could also be detected in the circulation of nude mouse after transplanted with keratinocytes expressing protein [22]. The big issue is very little factor IX that actually reaches the systemic circulation. The level of factor IX was only 0.5 ng/ml to 3 ng/ml from day 1 to day 7 after transplantation. The authors estimated that the efficiency of factor IX secreting into circulation is only 2.6% [22]. The similar conclusion came from a study in keratin-promoter transgenic mice [21]. It is estimated that a graft of 25–30% total surface area is needed to achieve 2% of normal physiological level (100 ng/ml) of factor IX [21]. Although epidermal transgene expression may not be practicable for hemophilic diseases which need medium to large amount of gene production ( µg/ml to mg/ml) for replacement, it may be feasible for systemic disorders such as a deficiency of growth hormone (GH), adrenocorticotropic hormone or parathyroid hormone, in which the physiological range is pg/ml to ng/ml. In nude mice grafted with keratin-promotor-driven GH transgenic mouse skin, 0.1–0.4 ng/ml of human GH could be detected in the bloodstream [23]. Considering the physiological level of human GH is 0 to 5 ng/ml, it is possible to achieve this level by an increase of the graft size or the area of GET-treated skin.

One advantage of skin GET with MEA is that the level of gene expression is controllable by adjusting the treated area. We demonstrated that the gene production could be significantly increased proportionally by extension of GET area. To achieve this goal, we can either treat more areas with same MEA or expand our current MEA to larger size without change of the pin gap so that the same parameters can be applied to this modified MEA. In addition, our group is investigating other factors may enhance the efficiency of GET or facilitate gene product to diffuse into circulation meanwhile without cause of cell toxicity. So the disadvantage of low level of gene product in the blood may be overcome.

In conclusion, this is the first study utilizing a HLGP model with skin features similar to human skin to characterize the GET with a non-invasive MEA electrode. Efficient gene delivery with an increase up to 4 logs can be achieved by GET with the MEA. After skin GET with the MEA, exclusively epidermal expression was observed, and high level gene expression can be maintained for up to 12–15 days. We observed that skin changes in HLGP caused by GET with the MEA are minimal and milder than those in normal hair guinea pig. However, only a small portion of gene product reached the systemic circulation of the animal. These results suggest skin gene delivery with our approach can be a safe, efficient, non-invasive method for skin disorders, vaccinations and possibly systemic diseases with physiological levels that are in the range of pg/ml to ng/ml, but may not be suitable for conditions requiring a larger amount of gene product.
